# Management of diabetic foot ulcers: polyherbal formulations and novel delivery systems for herbal active components

**DOI:** 10.3389/fphar.2025.1648540

**Published:** 2025-10-03

**Authors:** Bowen Feng, Jinjin Dou, Yongji Li, Yihan Wang, Changmei Wu, Xiaodong Lan, Yi Su, Xiwu Zhang

**Affiliations:** ^1^ Graduate School, Heilongjiang University of Chinese Medicine, Harbin, China; ^2^ The Four Hospital of Heilongjiang University of Chinese Medicine, Heilongjiang University of Chinese Medicine, Harbin, China

**Keywords:** diabetic foot ulcers, polyherbal formulations, herbal medicines, novel drug delivery systems, regenerative medicine, herbal active components

## Abstract

Diabetic foot ulcers (DFUs) represent devastating complications associated with high risks of amputation and mortality. The complex pathophysiology of the wound microenvironment and systemic involvement render conventional therapies costly and limited in effectiveness. Consequently, there is an urgent demand for the development of effective and comprehensive therapeutic and management strategies. Herbal medicines and their formulations can transform DFU management from passive debridement to active regeneration through multi-target synergy and holistic intervention, offering low-cost and safe alternatives. Integrating herbal active components with polymeric materials enables the development of novel delivery systems and tissue-engineered scaffolds. This interdisciplinary approach is emerging as a promising therapeutic strategy in regenerative medicine and dermal engineering. This paper compares cellular healing mechanisms in DFUs and normal wounds, elucidates the mechanisms of action of herbal medicines and their formulations in treating DFUs, and synthesizes the applications of novel delivery systems for herbal active components in DFU therapy.

## 1 Introdution

Diabetes, a formidable global healthcare challenge, without global intervention, over 1.31 billion people are projected to live with diabetes by 2050 (GBD 2021 Diabetes Collaborators, 2023). Among them, DFUs, one of the most dangerous complications of diabetes, remain a major risk factor for disability and death today (GBD, 2021, Diseases and Injuries Collaborators, 2024). Currently, the clinical outcomes of DFUs are unsatisfactory, with approximately one million people worldwide facing amputation each year and poor patient prognosis (Lan et al., 2023). This results in expenditure on DFU wound management far exceeding that on hospitalization (Nussbaum et al., 2018). The global market for trauma treatment products had already reached $12 billion in 2020 and is expected to reach $18.7 billion by 2027, growing at a rate of 40 percent (Sen, 2021).

During DFU wound healing, the hyperglycemic environment causes microcirculatory impairment, while the immunosuppressive state increases infection with multidrug-resistant organisms (MDROs), thus forming an “ischemia-infection-inflammation” vicious cycle. This local immune imbalance ultimately triggers gangrene or even amputation (Barman and Koh, 2020; Nirenjen et al., 2023; Ju et al., 2024a; Zhang H. et al., 2025). Consequently, impaired wound healing in DFUs represents not only a localized tissue defect but also a systemic, life-threatening crisis requiring multidimensional interventions to disrupt this pathological cycle (Kim et al., 2024). Internationally, the standard DFU treatment modalities mainly include glycemic control, infection control, debridement and drainage, decompression therapy, wound dressings, and individualized therapy (Bus et al., 2024b). The International Working Group on the Diabetic Foot (IWGDF) states that the severity of the diabetic foot is largely related to differences in the standard of care for the foot (Bus et al., 2024a). While debridement and negative pressure therapy remain indispensable for severe ulcers, clinical options for DFUs remain limited. Prevention and comprehensive management therefore constitute critical strategies for reducing amputation rates, recurrence, and mortality (Jeffcoate et al., 2024).

Herbal therapies were positioned as both naturopathic and complementary approaches in global medical contexts (Liu et al., 2022; von Schoen-Angerer et al., 2023). Unlike conventional therapies focused on single-target interventions, herbal therapies provide holistic modulation of DFUs. Modern research has transformed herbal therapy from an “empirical medicine” perception to evidence-based, multi-target interventions with clarified mechanisms. Key therapeutic actions include anti-inflammatory effects, oxidative stress inhibition, angiogenesis promotion, and epithelial regeneration (Jarić et al., 2018; Michalak, 2023). These characteristics facilitate a shift from passive debridement to active tissue regeneration in DFUs. Notably, ON101—the world’s first natural drug targeting macrophage polarization—has been approved by the National Medical Products Administration (NMPA). In Phase III multiregional clinical trials (MRCTs) across the U.S. and China, DFU patients treated with ON101 achieved a 60.7% wound healing rate, significantly outperforming the 35.1% rate observed in control groups using absorbent dressings (Huang et al., 2021). Furthermore, active components from Chinese herbal medicines are being integrated with advanced pharmacological technologies to develop novel drug delivery systems. These systems enable targeted delivery, controlled release, reduced dosing frequency, and enhanced patient compliance while optimizing therapeutic efficacy (Kim et al., 2019; Qadir et al., 2021).

This paper initiates a comparative analysis of the dynamic cellular mechanisms underlying normal wound healing *versus* impaired wound healing in DFUs. From a molecular perspective, we provide a scientific rationale for the multi-target synergistic effects of herbal medicines and their formulations in treating DFUs. Additionally, we summarize a burgeoning research trend: the integration of herbal active components with novel drug delivery systems for DFU therapy.

## 2 The complex pathophysiology of difficult-to-heal wounds in DFUs

Previous research has primarily focused on the multifactorial etiology of DFUs, including neuropathy, vasculopathy, and infection. However, recent studies highlight the dynamic regulation of DFU wound healing, with cellular-level interventions gaining prominence as a key research frontier. Emerging evidence underscores the critical role of cellular mechanisms and molecular signaling pathways in modulating DFU repair processes.

### 2.1 The differences in cellular mechanisms between normal wound healing and impaired wound healing in DFUs

The wound healing cascade is a precisely orchestrated and meticulously executed biological process, encompassing numerous biochemical and cellular responses that are regulated by the body’s immune system to restore the integrity of skin and subcutaneous tissues. It is divided into four highly interrelated and partially overlapping phases: hemostasis, inflammation, proliferation, and remodeling (Yuan et al., 2023) In diabetic patients, underlying pathological factors such as neuropathy, peripheral artery disease, and persistent hyperglycemia disrupt the normal wound repair process, leading to stalled healing and increasing vulnerability to infection. Once infection occurs, it combines with oxidative stress and chronic inflammation triggered by excessive reactive oxygen species (ROS), further exacerbating tissue damage, infection severity, and ulcer formation/progression. These interconnected mechanisms form a vicious cycle that drives non-healing wounds to ultimately culminate in DFUs (Dayya et al., 2022; Deng et al., 2023).

**FIGURE 1:**
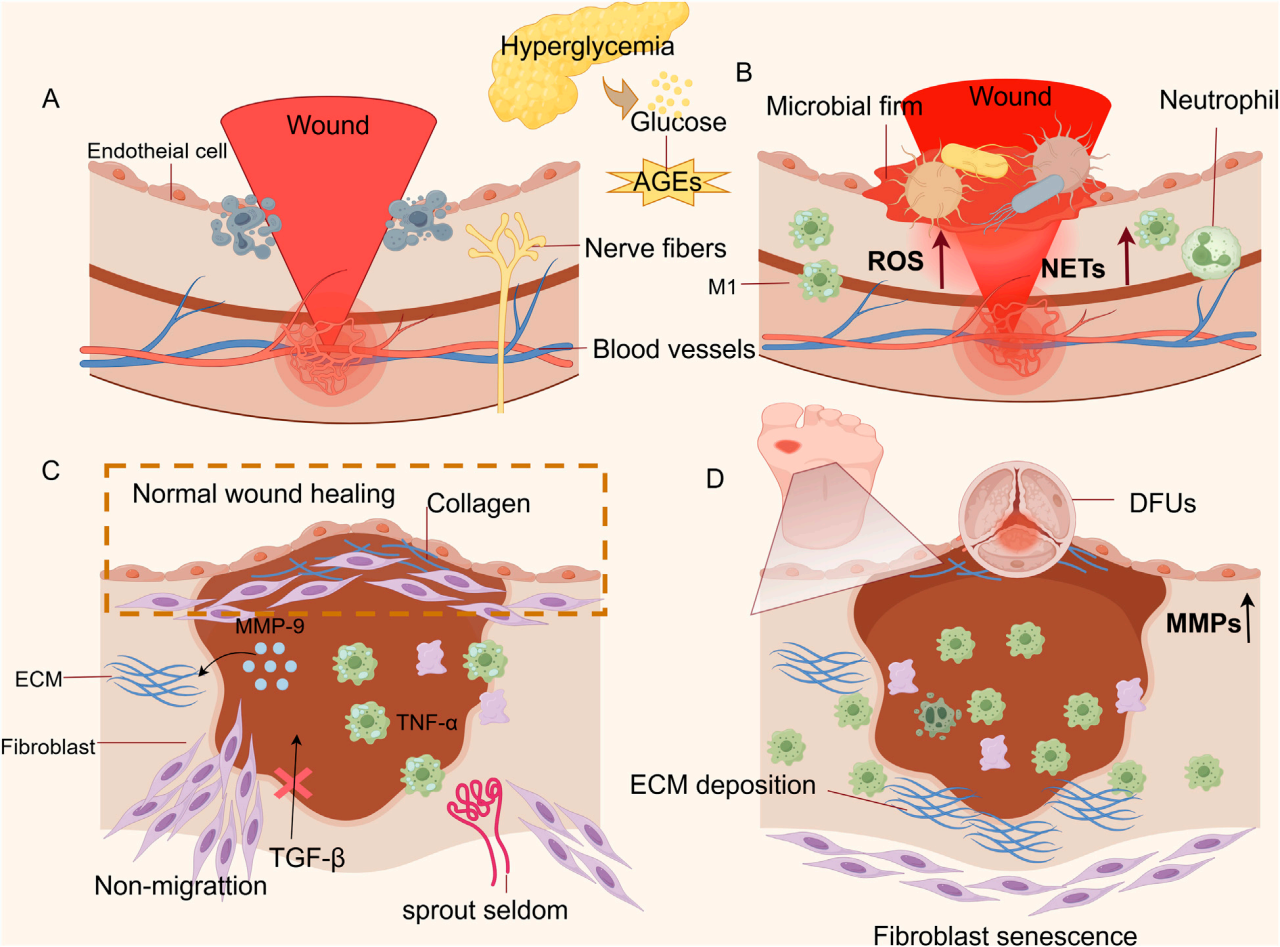
Diagram of the cellular mechanism of non-healing of DFU wounds. **(A)** Impaired hemostatic phase: There is an impairment in the ability of platelets to aggregate at the wound site, accompanied by their excessive activation, which leads to thrombus formation and an inability to clear necrotic cellular debris from the wound. **(B)** Prolonged inflammatory phase: The overproduction of ROS and NETs, coupled with the persistent presence of M1 macrophages, establishes a vicious cycle between oxidative stress and inflammation. **(C)** Shortened proliferative phase: Unlike normal wounds, DFUs fail to achieve epithelial regeneration. Fibroblast migration is impaired, and angiogenesis is reduced. **(D)** Impaired remodeling phase: Keratinocyte migration is compromised, resulting in abnormal epidermal migration and incomplete wound closure; the sustained high levels of MMPs cause excessive degradation of the ECM, hindering collagen remodeling and leading to difficulties in DFU wounds healing. (Thanks for Fig draw).

The hemostatic phase commences immediately after injury and may persist for several hours, aiming to control bleeding and restrict systemic microbial dissemination. The body initiates primary hemostasis first, during which vasoconstriction occurs instantaneously, and platelets are activated and aggregated to form a provisional platelet plug. Secondary hemostasis begins with the activation of the coagulation cascade, generating thrombin that catalyzes the conversion of fibrinogen to fibrin. The cross-linking of fibrin into a mesh that envelops the platelet aggregate, culminating in the formation of a stable clot, signifies the conclusion of the hemostatic phase (Wilkinson and Hardman, 2020; Singer, 2022). Subsequently, activated platelets release growth factors including platelet-derived growth factor (PDGF) and transforming growth factor-beta (TGF-β), which recruit inflammatory cells such as neutrophils to infiltrate the wound site. This cellular infiltration signifies the commencement of the inflammatory phase of healing (Jarić et al., 2018; Szułdrzyński et al., 2020). In DFUs, healing impairment originates from hemostatic dysfunction (Figure 1A). Hyperglycemia disrupts platelet aggregation, while advanced glycation end-products (AGEs) suppress fibrinogen function, impairing fibrin-platelet cross-linking and reducing clot stability. Concurrently, platelet hyperactivation promotes microthrombosis and hypoperfusion, dysregulating the coagulation cascade (Gawlowski et al., 2007; Brings et al., 2017; Hicks and Selvin, 2019; McDermott et al., 2022).

The inflammatory phase initiates almost concurrently with hemostasis, typically spanning 1–4 days post-injury. However, its duration may extend to weeks or even years due to underlying pathologies (Tomic-Canic et al., 2020). During this stage, neutrophils may recruit monocytes by secreting several inflammatory factors (IL-1, IL-6, IL-8, IL-10) and the chemokine MCP-1 to regulate the inflammatory response (Singer, 2022; Peña and Martin, 2024). Subsequently, infiltrating monocytes differentiate into M1 macrophages (the predominant cell type during periods of inflammation.) M1 macrophages exert antimicrobial activity primarily by recognizing pathogen-associated modifier proteins (PAMPs) and damage-associated modifier proteins (DAMPs) and by releasing NO, ROS, and pro-inflammatory cytokines to clear pathogens and cellular debris. Ultimately, the sign of inflammation subsiding is the clearance of neutrophils through phagocytosis by macrophages or re-entry into the vascular system (Hassanshahi et al., 2022; Sharifiaghdam et al., 2022). As shown in Figure 1B, whereas the key factor contributing to the delayed healing of DFUs wounds is the persistent overpresence of M1 macrophages and their subsequent inability to convert to the M2 repair phenotype, which amplifies the stimulatory response to cytokines (Aitcheson et al., 2021; Tang et al., 2022). Under hyperglycemic conditions, neutrophils continuously release ROS and stimulate the upregulation of peptidyl arginine deaminase 4 (PAD4), which leads to the overproduction of neutrophil extracellular traps (NETs) and damage to tissue cells (Yang et al., 2023; Zhang Y. et al., 2025). This activates pathways such as NF-κB and promotes the release of inflammatory factors, resulting in local microcirculation disorders that further lead to increased inflammation and vascular and neurological tissue lesions in the ulcerated area (Li et al., 2023; Huang W. et al., 2020; Yang et al., 2020). Important at this stage is an impaired immune response, which will not prevent colonization by pathogenic bacteria, and an excess of AGEs will lead to microbial membrane formation (Liu C. et al., 2020; Xie et al., 2020). In addition, AGEs bind to receptors for advanced glycation end products (RAGE)on various cell types, activating multiple signaling pathways including MAPK/ERK, TGF-β, JNK, and NF-κB. This process increases intracellular oxidative stress, exacerbates local inflammatory responses, and ultimately impedes the transition to subsequent healing phases (Huijberts et al., 2008; Khalid et al., 2022). How to dynamically regulate the timing of the transition from the inflammatory to the proliferative phase is a current difficulty in the treatment of DFUs (Zhou et al., 2022). Concurrently,the disruption of endogenous antioxidant defense mechanisms constitutes a critical pathogenic factor in the development of DFUs (Deng et al., 2021).

After hemorrhage and inflammation are controlled, a proliferative phase, which is longer and can last for several weeks, takes place. The beginning of the proliferative phase is marked by the migration of fibroblasts into the wound, proliferation, and secretion of extracellular matrix (ECM) components to build granulation tissue as a new cellular scaffold (Raziyeva et al., 2021). Angiogenesis is another important process in this phase, where the macrophage phenotype changes from an inflammatory state (type M1) to a healing state (type M2), releasing healing cytokines: PDGF, epidermal growth factor (EGF), and vascular endothelial growth factor (VEGF), which promote deposition of the ECM and repair of broken blood vessels and formation of new blood vessels (Peng et al., 2020). Keratinocytes ultimately achieve epithelial regeneration by secreting local growth factors TGF-α, TGF-β, and keratinocyte growth factor (KGF/FGF 7), as well as by being influenced by mechanical factors (“free edge effect”) (Huang et al., 2023). As shown in Figure 1C, in DFU wounds, the persistence of M1 macrophages causes excessive release of TNF-α and promotes the release of Matrix Metalloproteinase-9 (MMP-9) rom keratinocytes to continuously degrade the ECM, which impairs the migratory ability of keratinocytes and thus prevents the re-epithelialization of the ulcerated wounds (Deng et al., 2024). High accumulation of AGEs leads to fibroblast senescence and is not regulated by the migration stimulator TGF-β, inhibiting their migration into the wound (Deng et al., 2023; Huang et al., 2023). Oxidative stress from hyperglycemia-induced ROS surge elevates oxygen consumption, suppressing hypoxia-inducible factor-1α (HIF-1α) and downstream VEGF expression. This disruption of angiogenic signaling cascades culminates in reduced neovascularization, perpetuating microcirculatory deficits in ulcerated tissues (Catrina and Zheng, 2016).

The remodeling phase is the final stage of wound healing and can last from weeks to years after injury. Fibroblasts upregulate the expression of type I collagen, and macrophages release MMPs into the deposited ECM, causing wound contraction and tissue remodeling through protein hydrolysis and endocytosis. Furthermore, as the ECM subsides, the dense connective tissue is replaced by newborn natural tissue, maintaining normal tissue function of the skin (Sutherland et al., 2023). Finally, as apoptosis of vascular cells and myofibroblasts heralds the end of healing, the conversion of cell-rich granulation tissue into collagen-filled cells results in scarless healing (Eckes et al., 2010; Reinke and Sorg, 2012). As shown in Figure 1D, during the tissue remodeling phase of DFUs, the persistent high-glycemic environment and excessive inflammatory response resulted in high expression of MMPs, leading to excessive degradation of ECM and stagnation of collagen remodeling (Fu et al., 2022). As shown in Figure 1D, persistent ROS and an inflammatory microenvironment induce premature senescence of fibroblasts. On one hand, this significantly inhibits their ability to differentiate into myofibroblasts, preventing the wound from actively contracting. On the other hand, it severely impairs collagen synthesis function, resulting in an insufficient total amount of newly synthesized collagen (Darby et al., 2014). Meanwhile, the accumulation of AGEs disrupts collagen fibers through irreversible covalent cross-linking, causing abnormal cross-linking and excessive degradation of the ECM. This leads to the inability of myofibroblasts to arrange properly at the wound site, ultimately resulting in wound closure failure (Asadipooya and Uy, 2019; Yang Y. et al., 2024).

### 2.2 Risk factors for difficult wound healing in DFUs

Multiple biological responses affect wound healing in DFUs, including persistent inflammation, oxidative damage, impaired angiogenesis, apoptosis, and aberrant ECM deposition. Among these, the vicious cycle formed by mutual reinforcement of oxidative stress and chronic inflammation is identified as a primary risk factor for delayed wound healing in DFUs (Jeffcoate et al., 2024; Xiao F. et al., 2024; Da Silva et al., 2025). Following organismal injury, a modest inflammatory response is a key defense mechanism to clear pathogens and initiate repair (Huang et al., 2022). However, insufficient inflammation weakens the skin’s antimicrobial barrier and increases the risk of infection. The loss of the natural barrier makes it easier for microorganisms outside the wound to invade the skin tissue and trigger inflammation (Uberoi et al., 2024; Zielińska et al., 2023). Conversely, DFUs often exhibit a hyperinflammatory microenvironment marked by excessive pro-inflammatory cytokine release and ROS overproduction. These factors not only induce oxidative cellular damage but also promote local ischemia and hypoxia, further exacerbating ulcer progression. Importantly, diabetic wounds are frequently prone to infection, which is closely associated with biofilm formation and persistent inflammation. Compared to ordinary chronic wounds, pathogenic microorganisms on DFU wounds exhibit a higher propensity to form biofilms, thereby inducing excessive inflammation. Moreover, patients with DFUs suffer from impaired immune responses, leading to microbial dysbiosis in the wound microenvironment (Jnana et al., 2020a; Mudrik-Zohar et al., 2022). The core mechanism involves biofilm formation by multiple pathogens. The microbial communities in DFUs predominantly consist of Gram-positive and Gram-negative bacteria, including *Staphylococcus aureus*, *Streptococcus* spp., and *Pseudomonas aeruginosa*. Among these, the biofilm structures of *S. aureus* and *P. aeruginosa* can physically impede immune cell infiltration. More critically, once microbial biofilms mature, the embedded microorganisms exhibit remarkable resistance and evasion capabilities (evading phagocytosis and clearance by immune cells), posing a fundamental barrier to sustained wound infection and impaired healing (Pouget et al., 2020; Lou et al., 2025).

Currently, the treatment of choice for DFU infections is antibiotic therapy. However, due to the limitations of the antibiotic antimicrobial spectrum, the cost of treatments aimed at killing all microorganisms in the wound is significant and is detrimental to wound healing in DFUs (Jnana et al., 2020b; Liu C. et al., 2020). A recent clinical study supports this notion: metagenomic shotgun sequencing analysis of wound microbiomes from 100 DFU patients revealed that antibiotic therapy struggles to address the complex polymicrobial-multidrug-resistant pathological microenvironment. In contrast, debridement surgery promotes healing by dynamically modulating the wound microbiome to increase the proportion of commensal bacteria (Kalan et al., 2019). Therefore, enhancing healing may be achieved by dynamically regulating the DFU wound microbiome to augment beneficial bacteria while reducing pathogenic bacteria, rather than attempting to eliminate all bacterial species from the wound.

## 3 Herbal medicines and their formulations for the treatment of DFUs

Herbal medicines and their formulations can exert multi-targeted effects to treat DFUs, mainly including inhibition of inflammatory response, inhibition of oxidative stress, promotion of angiogenesis, epithelial regeneration, and other various effects. The pathways can be summarized as the Wnt-βcatenin, Notch, and NF-κB pathways that regulate inflammation, the Nrf 2/ARE and MAPK pathways that inhibit oxidative stress, and the PI 3K/Akt, TGF-β/Sma, and HIF-1α/VEGF pathways that promote angiogenesis and epithelial regeneration (Zhou et al., 2022). The persistent vicious cycle of oxidative stress and chronic inflammation, coupled with impaired angiogenesis, remains a critical therapeutic target in managing DFUs. Based on dynamic phase-specific regulation of DFU pathogenesis, this study categorizes the multi-target effects of commonly used traditional Chinese medicine (TCM) empirical formulas and classical prescriptions into two synergistic mechanisms: “anti-inflammatory-antioxidative” actions during the inflammatory phase, and “pro-angiogenic-epithelial regenerative” effects during the remodeling phase.

### 3.1 Anti-inflammatory - inhibits oxidative stress

#### 3.1.1 San Huang Xiao Yan recipe

San Huang Xiao Yan recipe (SHXF), a commonly prescribed TCM empirical formula for DFUs in Chinese hospitals, comprises *Rheum palmatum* L., *Scutellaria baicalensis* Georgi, *Crataegus pinnatifida* Bunge., *Phellodendron chinense* C.K.Schneid., *Isatis tinctoria* L., *Forsythia suspensa* (Thunb.) Vahl, *Polygonum bistorta* L., *Commelina communis* L., *Glycyrrhiza uralensis* Fisch., *Coptis chinensis* Franch., and *Polygonum cuspidatum* var. Spectabile Noter.

In a clinical trial involving 86 DFU patients, researchers demonstrated that SHXF combined with silver dressings significantly reduced infections caused by MDROs (Chen et al., 2023). Another trial with 98 DFU patients showed that SHXF combined with yellow horse tincture downregulated matrix metalloproteinase-2 (MMP-2) expression, upregulated tissue inhibitor of metalloproteinases-1 (TIMP-1), and markedly reduced ulcer size (Zhao L. et al., 2021). The remarkable clinical efficacy of SHXF is attributed to its exertion of a multi-target regulatory mechanism for the treatment of DFUs.

Using streptozotocin (STZ)-induced DFU models in C57 mice and Sprague-Dawley (SD) rats, proteomic analysis of skin wounds revealed that SHXF significantly reduced levels of the inflammatory protein high mobility group box 1 (HMGB1), effectively blocking HMGB1-mediated inflammatory responses and decreasing inflammatory cell infiltration at wound sites. Immunofluorescence analysis further showed that SHXF activated AMP-activated protein kinase (AMPK) pathway phosphorylation, promoted nuclear translocation of the transcription factor Nrf2, and enhanced expression of the downstream antioxidant enzyme heme oxygenase-1 (HO-1). This dual anti-inflammatory and antioxidative regulatory mechanism ameliorated oxidative stress injury, reduced the wound’s inflammatory microenvironment, and ultimately facilitated neovascularization and epithelial remodeling (Zhang et al., 2023c). Additionally, a study revealed that SHXF exerts immunomodulatory effects by improving the trauma microenvironment. Animal experiments confirmed that SHXF regulates CD4^+^ T cells and attenuates immune-inflammatory responses. Simultaneously, SHXF inhibited STAT3 phosphorylation and suppressed Th17 cell activation and interleukin-17 (IL-17) secretion, thereby promoting DFU wound healing through immune modulation and reduction of excessive inflammatory responses. Furthermore, using network pharmacology and molecular docking, the active ingredients in SHXF predicted to be effective against DFUs were identified as β-sitosterol, wogonin, 7-methoxy-2-methyl isoflavone, formononetin, baicalein, isocorypalmine, (S)-canadine, vestitol, shinnerocarpin, and (R)-canadine (Deng et al., 2025).

#### 3.1.2 Ruyi Jinhuang powder

Ruyi Jinhuang powder is a classic formula from the Ming Dynasty in China, included in the Surgical Zhengzong written by Chen Shikong. Its specific medicinal flavor composition is *Curcuma longa* L., *Rheum palmatum* L., *Phellodendron chinense* C.K.Schneid., *Atractylodes lancea* (Thunb.) DC., *Magnolia officinalis* Rehder. et Wils., *Citrus reticulata* Blanco, *Glycyrrhiza uralensis* Fisch, *Arisaema erubescens* (Wall.) Schott, *Angelica dahurica* (Fisch ex Hoffm.) Benth. and Hook. f., and *Trichosanthes kirilowii* Maxim.

In a single-center clinical trial, combination therapy with moist exposed burn ointment (MEBO) and Ruyi Jinhuang powder achieved up to 92% efficacy in healing DFUs (Zhan et al., 2021)**.** The core mechanism can be summarized as synergistic regulation of the DFU microenvironment through “anti-inflammatory and oxidative stress inhibition.” Ruyi Jinhuang powder reduced the production of ROS in high glucose-treated human skin fibroblasts (HDF-a) and attenuated cellular oxidative stress in a dose-dependent manner. Quantitative PCR (qPCR) results demonstrated significant downregulation of inflammatory cytokines, including IL-1α, IL-1β, and IL-6, with the treated group showing only 50% of the IL-6 levels observed in the control group, indicating robust anti-inflammatory activity. Further in vivo experiments confirmed that Ruyi Jinhuang powder promoted wound angiogenesis by upregulating CD31 and VEGF-A expression, achieving basic wound closure after 14 days of treatment (Wu et al., 2022). Electron microscopy revealed recovery of fibroblasts and neuronal cells, maturation of granulation tissue, and neutrophil infiltration in the treated group of DFU rats. Through molecular docking and KEGG pathway enrichment analysis, it was hypothesized that Ruyi Jinhuang powder alleviates diabetic peripheral neuropathic pain by activating neuroactive ligand-receptor interactions in the PI3K-Akt signaling pathway and inhibiting the FoxO signaling pathway. Further, network pharmacological analysis revealed that the DFU-treating components of Ruyi Jinhuang powder were luteolin, trans-caryophyllene, ar-turmerone, palmitic acid, methyl palmitate, gallic acid, demethoxycurcumin, berberine, and rhein (Li X.-Y. et al., 2022).

#### 3.1.3 Huangbai liniment

Huangbai liniment is a widely used clinical formula in China, composed of *Forsythia suspensa* (Thunb.) Vahl, *P. chinense* C.K.Schneid., *Lonicera japonica* Thunb., *Taraxacum sect.* Taraxacum F.H.Wigg. , and *Scolopendra subspinipes mutilans* L. Koch.

In a multicenter clinical trial of 720 patients with DFUs, the overall efficacy of Huangbai liniment treatment was demonstrated to be 96.48% with no adverse events within 4 weeks of treatment (Liu Y. et al., 2020). Another study demonstrated that compared to antimicrobial calcium alginate wound dressings, Huangbai Liniment exhibits superior antimicrobial efficacy while offering greater economic advantages (Yang G. et al., 2024).

Basic studies have elucidated the molecular mechanisms underlying its multi-targeted therapeutic effects on DFUs. The Huangbai liniment upregulates the anti-inflammatory factor TGF-β1 and significantly downregulates downstream pro-inflammatory targets, including IL-6, IL-1β, MMP-9, CXCL-1, and CCL2, while inhibiting the IL-17A pathway to exert anti-inflammatory effects (Zhang et al., 2022). Critically, it ameliorates the oxidative stress microenvironment of DFUs by elevating total antioxidant capacity (T-AOC), superoxide dismutase (SOD) activity, and glutathione (GSH) levels, while reducing oxidative damage markers such as malondialdehyde (MDA) and 8-hydroxydeoxyguanosine (8-OHdG). This is achieved by upregulating the downstream antioxidant factor NQO1 and reversing the nuclear translocation of the transcription factor Nrf2, thereby activating the Nrf2 signaling pathway (Zhang et al., 2020). This dual regulatory mechanism of anti-inflammatory and oxidative stress inhibition enables Huangbai Liniment to achieve favorable therapeutic outcomes in treating DFUs.

### 3.2 Pro-angiogenic-epithelial regeneration

#### 3.2.1 Sheng-Ji Hua-Yu formula

Sheng-ji Hua-yu formula is an empirically established formula widely utilized in clinical practice in China, consisting of *Astragalus membranceus* (Fisch) Bunge, *Salvia miltiorrhiza* Bunge, *R. palmatum* L., *Daemonorops draco* (Willd.) Blume, *Arnebia euchroma* (Royle) I.M. Johnst., *A. dahurica* (Hoffm.) Benth. and Hook. f.ex Franch. and Sav.,*Hyriopsis cumingii* (Lea), and Calamine.

Basic experimental studies demonstrated that the Sheng-ji Hua-yu Formula downregulates activin/follistatin levels, inhibits downstream Smad2 phosphorylation and nuclear translocation, and facilitates keratinocyte migration, ultimately achieving wound re-epithelialization. Additionally, reduced pSmad2 levels suppressed TGF-β1-mediated fibrotic signaling and NF-κB nuclear translocation, thereby inhibiting inflammatory cytokine release from keratinocytes and promoting ECM remodeling (Kuai et al., 2018). Another study demonstrated that it promotes the phosphorylation of cyclic adenosine 3′,5′-monophosphate (cAMP) response element-binding protein (CREB), translocates activated protein kinase A (PKA) into the nucleus, upregulates the expression of the downstream angiogenic factor VEGF, and thereby promotes the transformation of pathological blood vessels into healthy neovascularization (Wang G. et al., 2025).

Further biomarker analyses in a DFU mouse model suggested that the Sheng-ji Hua-yu formula synergistically upregulates dopaminergic receptors (DRD1/DRD4) and downregulates cAMP to inhibit Nod-like receptor protein-3 (NLRP3) and CXCR4 expression, thereby modulating inflammation and oxidative stress. Concurrently, it increases angiotensin II type 1 receptor (AGTR1) and δ-opioid receptor (OPRD1) expression, driving dynamic balance between neuropeptides and vasoactive factors through neuroactive ligand-receptor interactions. This dual regulatory mechanism aims to resolve the vascular and neuropathic lesions impeding DFU wound healing (Jiang et al., 2021; Xiang et al., 2021; Ru et al., 2022).

#### 3.2.2 Huiyang Shengji decoction

Huiyang Shengji decoction represents a traditional Chinese medicine formula that has been refined through decades of extensive clinical practice across China. Its composition consists of Sinapis alba L. *Astragli Radix*, *Atractylodis Macrocephalae Rhizoma*, *Poria, Atractylodis Rhizoma*, *Cinnamomi Cortex*, *Aconiti Lateralis Radix Praeparata, Cervi Cornu Degelatinatum, Chaenomelis Fructus, Sinapis Semen, Rehmanniae Radix Praeparata, Angelicae Dahuricae Radix*, and *Glehniae Radix*.

In a clinical trial involving 42 patients with diabetic foot ulcers (DFUs), those treated with Huiyang Shengji decoction combined with Rehabilitation New Liquid achieved an overall efficacy rate of 100% (Huang and Zeng, 2020a). Recent studies on the mechanism of Huiyang Shengji decoction have gradually unveiled the mystery behind its remarkable therapeutic effect—a multi-mechanism co-regulation. On one hand, Huiyang Shengji decoction can synergize the processes of “angiogenesis and epithelial regeneration” to promote wound healing in DFUs. It promotes epithelial regeneration by inducing the secretion and expression of VEGF and increasing the level or activity of epidermal growth factor receptor (EGFR), a key receptor in keratinocytes. This, in turn, activates its downstream the PI3K/AKT signaling pathway (Liu et al., 2021). On the other hand, Huiyang Shengji decoction modulates the immune microenvironment by activating peroxisome proliferator-activated receptor γ (PPARγ), thereby regulating macrophage polarization, ROS production, and controlling NLRP3 inflammasome activation. This inhibits STAT3 phosphorylation and blocks its synergistic interaction with NF-κB, thereby exerting anti-inflammatory effects. Further network pharmacological analysis revealed that the key therapeutic constituents of Huiyang Shengji decoction for treating DFUs are calycosin, calycosin-7-glucoside, hypaconitine, and sinapic acid (Chen et al., 2025; Lin et al., 2025).

#### 3.2.3 Gubu decoction

Gubu decoction is from the famous Qing Dynasty physician Chen Shiduo’s “Record of Discriminating Evidence”, which consists of *Scutellaria baicalensis* Georgi, *Dendrobium nobile* Lindl., *Angelica sinensis* (Oliv.) Diels, *L. japonica* Thunb., *Glycyrrhiza glabra* L., *Viola philippica* var. philippica, *Chrysanthemum indicum* L., *Panax ginseng* C.A.Mey, and *Achyranthes bidentata* Blume.

Gubu decoction downregulates HIF-1α and promotes neovascularization in ulcerated areas, thereby alleviating the hypoxic microenvironment of DFUs. On the other hand, by activating TGF-β1 signaling, it drives Smad3 phosphorylation and nuclear translocation, inhibits matrix metalloproteinase (MMP) overexpression, reduces extracellular matrix degradation, and accelerates granulation tissue proliferation and epithelial regeneration (Wang et al., 2025b). These two mechanisms synergistically form a “vascular reconstruction-epithelial regeneration” dual-effect system, ultimately enabling ulcer repair from microcirculation improvement to parenchymal tissue regeneration throughout the healing process. Another study demonstrated that Gubu Decoction directly promotes fibroblast growth factor 7 (FGF7) release by reducing miR-155 levels, significantly decreasing M1-type markers (iNOS, COX-2) and pro-inflammatory cytokines (IL-6, IL-1β), while enhancing DFU healing (Wang et al., 2025a). This reveals that it can play a synergistic role in regulating macrophage polarization and ameliorating the wound’s inflammatory microenvironment.”

**FIGURE 2:**
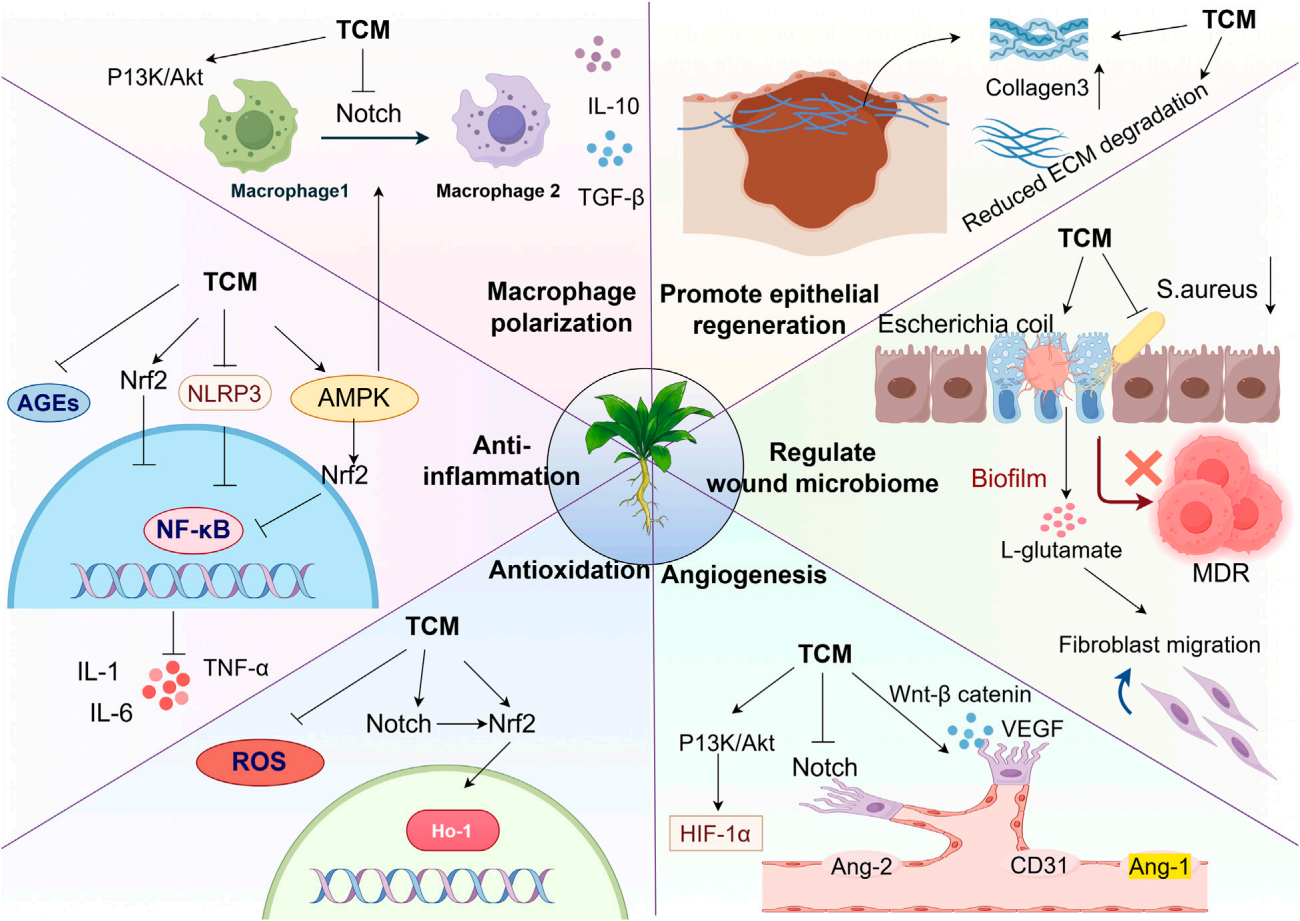
Multi-targeted effects of TCM in the treatment of DFUs: Regulating macrophage polarization: TCM activate the PI3K/Akt, Notch, Nrf2, and AMPK signaling pathways, promoting the transition of macrophages from the M1 to M2 phenotype and releasing pro-healing factors. Suppression of Inflammatory and oxidative cascades: TCM can block the nuclear translocation of NF-κB by inhibiting the accumulation of AGEs and ROS, as well as inhibiting the NLRP3 inflammasome pathway. This reduces the production of pro-inflammatory cytokines (IL-6, IL-10, TNF-α), thereby reducing inflammation and oxidative stress. Promoting epithelial regeneration and angiogenesis: TCM enhances type III collagen synthesis while ECM degradation, thereby accelerating epithelial cell repair. Concurrently, TCM activates the PI3K/Akt and Wnt/β-catenin signaling pathways while inhibiting the Notch pathway to exert pro-angiogenic effects. Modulation of the wound microbiome: Herbal medicines beneficially reduce the abundance of pathogenic bacteria and increase commensal bacteria in wounds without inducing antibiotic resistance, effectively rebalancing the wound microbiome (Thanks for Fig draw).

Tables 1, 2 summarize the pharmacological mechanisms and clinical evidence of other herbal medicines and their formulations for DFUs. Additionally, the multi-target mechanisms of action of TCM in treating DFUs are summarized in Figure 2.

**TABLE 1:** Multi-targeted therapeutic pathways of herbal formulations for the treatment of DFUs.

Herbal formulas	Composition	Prediction of active ingredients in therapeutic DFUs	Diabetes induction and excision wound model	Dosing time/mode of administration	Promoting healing mechanisms in DFUs	References
Jingfang ganules	*Nepeta cataria* L., *Saposhnikovia divaricata* (Turcz. ex Ledeb).Schischk., *Heracleum hemsleyanum* Diels, *Bupleurum chinense* DC., *Kitagawia praeruptora* (Dunn) Pimenov, *Poria cocos* (Schw). Wolf, *Platycodon grandiflorum* (Jacq). A. DC., *Hansenia weberbaueriana* (Fedde ex H. Wolff) Pimenov and Kljuykov, *Conioselinum anthriscoides*, *Citrus aurantium* L., and *Glycyrrhiza uralensis* Fisch.ex DC.	Scopolamine Lactone and Hesperidin	STZ-induced DFU rats (two full-thickness wounds of 2 cm in diameter on both sides of backs)	23 days/gavage (1or 2 g/kg/day)	1. Inhibition of oxidative stress-anti-inflammatory: increases SOD activity and reduces MDA, MPO and GSSG in rat tissues and downregulates the pro-inflammatory cytokines TNF-α and IL-1β 2. Promote angiogenesis: significantly increase CD31 expression 3. Regulation of blood glucose metabolism: compared with the control group, blood glucose was significantly reduced after treatment	Wang R. et al. (2025)
Zizhu ointment	Cinnabar, *Arnebia euchroma* (Royle ex Benth.) I.M.Johnst., *Daemonorops draco* Bl., and *Astragalus mongholicus* Bunge, ejiao, Borneolum	Isovalerylshikonin, mandenol, daidzein,kaempferol and formononetin	TypeⅡ Diabetic C57 BL/6 mouse Model (full-thickness skin wounds (1 × 1 cm with depth to the fascial layer on the back)	14 days/bandaged Zizhu ointment dressings	1. Inhibit inflammation: inhibit NLRP3 and IL-1β 2. Promote angiogenesis and tissue remodeling: Significantly increase the expression of collagenogenesis-related genes α-SMA, Collagen I, Collagen III, and VEGF in the wound 3. Regulation of macrophage polarization: activation of the PI 3 K/AKT signaling pathway to infiltrate M2 macrophages	Wang J. et al. (2022), Sun et al. (2025)
Si-Miao-Yong-An decoction	*Lonicera japonica*, *Scrophularia ningpoensis*, *Angelica sinensis*, and *Glycyrrhiza uralensis*	Quercetin and Kaempferol	Db/db mice (two 6-mm circular full-thickness wounds on the dorsum)	14days/receiving intragastric administration (20 g/kg/day)	1. Improving the local inflammatory microenvironment: inhibition of the AGE-RAGE signaling pathway; downregulate TNF, AKT 1, IL 6 and IL 1B 2. Promote macrophage polarization	Zhang et al. (2025d), 2025c
Huhuang decoction	*Reynoutria japonica* Houtt, *Astragalus mongholicus* Bunge, *Phellodendron chinense* C.K. Schneid, *Coptis chinensis* Franch, *Salvia miltiorrhiza* Bunge, *Paeonia veitchii* Lynch, and *Spatholobus suberectus* Dunn	Tetrahydropalmatine, emodin, rosmarinic acid, citric acid, berberine and cryptotanshinone	STZ-induced DFU rat model (four full-thickness defect wounds with diameters of 9 mm were created on the back)	12 days/alternating between oral and application (50 or 100 mg/mL)	1. Anti-inflammatory: inhibit NF-kβ pathway, downregulate IL-6, TNF-α and IL-1β expression 2. Promote angiogenesis: upregulate the expression of CD 31, HIF-1α and VEGF-α. 3. Promote vascular endothelial cell proliferation: enhanced expression of proliferation-related protein (cyclinD 1)	Zhang et al. (2025b)

**TABLE 2:** Multiple pathway mechanisms of herbal medicines for DFUs.

Herbal	Active ingredient	Diabetes induction and excision wound model	Dosing time/mode of administration	Mechanism	References
*Paeonia lactiflora* Pall	Paeoniflorin	STZ-induced DFU rats (a full-thickness skin wound with 5 mm diameter was created on the dorsal hind foot)	16 days/gavage once daily (15 or 30 mg/kg/day)	1. Anti-inflammatory: downregulate (IL)-1β, IL-18 and TNF-α, inhibit NF-κB pathway; downregulate downstream signaling chemokine receptor CXCL2, inhibit inflammation induced by NLRP3 signaling pathway 2. Inhibit oxidative stress: reduce ROS; upregulate glutathione peroxidase (GSH-px)	Huang et al. (2020c), Sun et al. (2021)
*Coptis chinensis* Franch.	Berberine	STZ-induced DFU rats (two wounds diameter, 2 cm were created on the back)	12 days/topical treatment	1. Inhibit oxidative stress: regulate TrxR 1/JNK signaling pathway, downregulate ROS level and MDA content 2. Promote ECM synthesis: downregulation of MMP 9, upregulation of TGF-β1 and TIMP 1	Zhou et al. (2021)
*Calvatia gigantea* (Batsch ex Pers.) Lloyd	Extracts	Leptin receptor-deficient db mice (1 cm2 wound on the dorsal area)	14 days/topical administration (100 μg/mL)	1. Regulate the microbiome: reduce the abundance of Staphylococcus aureus, enrich the beneficial bacterium Escherichia coli which can secrete L-glutamate to play a role in promoting the proliferation and migration of keratinocytes and fibroblasts. 2. Regulating macrophage polarization: increasing TGF-β secretion, inhibiting inflammation and promoting angiogenesis	Ding et al. (2024)
*Scutellaria baicalensis* Georgi	Baicalin	STZ-induced DFU rats (the thick skin of rectangular area 2 × 5 mm on the foot)	16 days/gavage (25、50 and 100 mg/mL)	1. Pro-angiogenesis: upregulation of angiopoietin Ang-1 2. Anti-fibrosis: increase TGF-β and SMAD2/3 mRNA expression 3. Inhibition of oxidative stress: upregulation of HSP27/p-HSP27 protein content protects wounds from hyperglycemic damage	Mao et al. (2021)
*Paeonia suffruticosa*	Paeonol	STZ-induced DFU rats (the deep fascia with a diameter of 1 cm was created on the back skin)	7 days/gavage (50 mg/kg/day)	1. Pro-angiogenesis: increased expression of angiogenic factors CD 31 and VEGF 2. Promotion of macrophage polarization: decreased expression of iNOS and activation of M2 macrophages 3. Anti-inflammation: decreased expression of IL-1β and TNF-α in traumatic inflammatory cells	Zhang et al. (2023b)
*Lonicera hypoglauca*	Lonicerin	STZ-induced DFU rats ((two full-thickness wounds (20 mm in diameter) were made on each side of the back))	20 days/gavage	1. Regulation of cellular autophagy: upregulation of Sirt 1 expression to improve the microenvironment of traumatic wound 2. Pro-angiogenesis: promote HUVEC migration and tube formation under oxidative stress	Lin et al. (2024)
*Bletilla striata* (Thunb. ex A. Murray) Rchb. f.	Bletilla striata Polysaccharide	STZ-induced DFU rats (two 6 mm excisional wounds on the dorsum)	12 days/topical administration once daily	1. Promote angiogenesis: regulate macrophage infiltration, downregulate TNF-α and IL-1β and CD 68 and CD 31 expression. 2. Anti-inflammatory-inhibit oxidative stress: inhibit NLRP 3 inflammatory vesicle activation and ROS production. 3. Improve insulin sensitivity.	Zhao Y. et al. (2021)
*Pueraria lobata* (Willd.) Ohwi	Puerarin	STZ-induced DFU mice (full-thickness wound)	10 days/gavage (120 mg/kg/day)	1. Anti-inflammatory: significantly downregulated F4/80, TNF-α, IL-1β and Cd 11b; inhibited the activation of NF-κB and MAPK pathways 2. Regulation of macrophage polarization: significantly increased IL-10, Arg-1, CD 206 and CD 163	Li et al. (2022a)
*Chebulae Fructus* Immaturus	Tannins	db/db DFU mice (the whole cortex wound with a diameter of 8 mm)	11day/topical administration	Promotes angiogenesis: upregulates the expression levels of HIF-1α and VEGF and CD 31	Qiu et al. (2024)
*Dragon’s blood*	Dracorhodin	STZ-induced DFU rats (a circular wound with a diameter of 1.5 cm wound on the spine	14 days/designatedtreatment	1. Inhibit inflammation and oxidative stress: downregulate TNF-α, IL-1 and hs-CRP; activate Nrf2 pathway to reduce ROS and lipid peroxidation. 2. Lowering blood sugar	Deng et al. (2022), Tang et al. (2025)

## 4 A novel delivery system of herbal active components for the treatment of DFUs

Based on previous studies, it is not difficult to find that the therapeutic effects of single components extracted and isolated from herbal medicines are often inferior to those of chemically synthesized drugs because the latter have enhanced targeted therapeutic properties through chemical modification. It is precisely because many of the active components of herbal medicines have limitations such as poor solubility, poor stability, low bioavailability, short half-life, and lack of targeting. These limitations severely restrict the application of herbal active components to the treatment of DFUs (Alavi et al., 2022). However, recent studies do not support this approach. The combination of herbal active components with novel delivery systems not only has a synergistic healing-promoting effect, but also enhances targeting and reduces toxicity.

### 4.1 Nanoparticles

Nanoparticles (NPs), as a primary type of modern drug delivery system, are typically based on natural or synthetic materials and form spherical aggregates with drugs through non-covalent interactions. Their proven manufacturing technology and inherent therapeutic properties demonstrate significant potential in DFU treatment. On one hand, nanoparticles are prepared *via* simple, controllable processes suitable for large-scale production. On the other hand, the functionalities of specific materials—as the antimicrobial properties of silver nanoparticles (Tripathi and Goshisht, 2022), antioxidant effects of cerium oxide nanoparticles (Xu et al., 2023), and broad-spectrum bacteriostatic activity of ZnO nanoparticles (Zhang et al., 2021)—can precisely target the core pathologies of DFU wounds, including inflammation and oxidative stress, thereby achieving a “carrier-treatment” dual advantage.

However, it is not recommended as a stand-alone therapeutic agent due to dose toxicity issues (Lewinski et al., 2008). Recent studies have designed a drug delivery system encapsulating herbal active components that not only retains their therapeutic efficacy but also exerts synergistic effects (Ai et al., 2024). It was found that by encapsulating Resina Draconis extract, which has wound-healing and hemostatic effects, and *Rhodiola rosea* L. extract, which has antioxidant and anti-inflammatory effects, in novel silver composite nanoparticles (AgNPs) with antimicrobial properties. Compared with the control group, the addition of herbal medicines significantly shortened the inflammatory period by decreasing the levels of inflammatory factors, and promoted angiogenesis by inhibiting LPO and increasing VEGF levels. And the loading of AgNPs was confirmed to significantly promote wound epidermal remodeling and granulation tissue repair compared with different dressing groups. This study reveals that herbal active components based composite nanomaterials can synergistically promote wound healing (Ju et al., 2024b). In another study, aqueous extracts of *Tagetes erecta* L. and *Portulaca oleracea* L. were encapsulated in AgNPs and further mixed with a polyherbal gel formulation. This herbal gel achieved 85% wound closure in diabetic rats and further demonstrated that the combination of AgNP-multi-herbal active components enhances antimicrobial activity while promoting DFU repair (Shelar et al., 2025).

### 4.2 Nanoenzymes

Nanoenzymes, with both nanomaterial properties and natural enzyme catalytic functions, are novel tools for regulating the microenvironment of DFUs. Nanoenzymes can play multiple synergistic roles in dealing with the complex pathological environment of DFUs: nanoenzymes with glucose oxidase (GOx) activity can break down glucose and improve the high-glucose environment (Liao et al., 2025); nanoenzymes mimicking catalase activity clear bacterial infections and inhibit wound microbial film formation (Zhao et al., 2023),Nanoenzymes with SOD- and CAT-like activities can reduce oxidative stress and improve wound ischemia and hypoxia by scavenging ROS (Xiao X. et al., 2024). Notably, the composite system of herbal active components and nano-enzymes can further realize the synergistic and synergistic effects of metabolic regulation and immunomodulation, providing a multidimensional intervention strategy for the treatment of DFUs.

In a recent study, metal-based nano-enzymes (MOFs) were combined with chlorogenic acid (CGA) extracted from *Lonicera japonica* Thunb and anchored in chitosan hydrogel to obtain a new type of nano-enzymatic hydrogel (MCGC). The results of the study showed that it could play a synergistic role, and the MOFs nano-enzyme and CGA released in the wound could play the catalytic activity of CAT-like enzyme to convert the accumulated H_2_O_2_ at the wound site into dissolved oxygen in the wound, alleviate the accumulation of ROS, and increase the oxygenation to promote wound healing. Moreover, CGA can clear the microbial membrane of the wound and reverse the bacterial infection of diabetic wounds (Wei et al., 2024). Another research group developed a novel delivery system for encapsulating astragalus polysaccharide (APS), an active ingredient of traditional Chinese medicine, in borax and iron-modified cerium nanoparticles (Fe/CeNP-PEG). It has been found that it can reduce inflammation by inhibiting the NLRP3/NF-κB signaling pathway, and animal experiments show that the healing rate can reach 97.6% (close to normal tissue). Moreover, the synergistic effect of APS and Fe/CeNP-PEG can dynamically regulate the microenvironment through antioxidant, anti-inflammatory, and pro-angiogenic effects, breaking through the limitations of traditional monotherapy (Zhang X. et al., 2025).

### 4.3 Exosomes

The latest breakthrough in treating DFUs is exosomes (MSCs-Exo) derived from mesenchymal stem cells (MSCs). One of the reasons for this is that MSCs have the ability to differentiate into a variety of cells that treat DFUs and secrete a variety of cytokines, growth factors, and chemokines that promote the healing of DFUs (Tienda-Vázquez et al., 2023; Wang H. et al., 2025).

Compared with MSC single-cell therapy, the proteins and signaling molecules on the surface of exosomes have strong targeting ability, which can transfer cytokines secreted by MSCs to target cells to regulate cell-to-cell signaling, promote immune response and tissue repair, and show multi-dimensional regulatory effects in wound healing (Bian et al., 2022; Sun et al., 2022). More importantly, adverse reactions such as immune rejection associated with transplantation of MSCs can be avoided (Wang H. et al., 2025). In recent years, studies on the combination of herbal active components and exosomes for wound healing in DFUs have gradually emerged. A recent study confirmed that pretreating mesenchymal stem cells (MSCs) with quercetin—a herbal active ingredient exhibiting anti-inflammatory and antioxidant properties—generated composite exosomes with significantly enhanced capacity to promote cell migration and ECM remodeling compared to untreated exosomes. Moreover, by 16S rRNA sequencing analysis, it was found that it could also play a role in promoting diabetic wound healing by regulating the intestinal microbiota (Wu et al., 2024). A recent study developed a novel vascular-targeting ginseng exosome loaded into a wireless biocompatible thermoelectric hydrogel. By mimicking endogenous electric field stimulation at wound sites, the system precisely controlled the release of pro-angiogenic ginseng exosomes, targeting electrophilic properties in epithelial and fibroblast cells to sustainably reverse endothelial dysfunction. Further experiments confirmed that this approach promoted tissue regeneration and revascularization in DFUs by activating the PPAR signaling pathway (Tan et al., 2025).

Currently, MSCs-Exo for the treatment of DFUs is still in the animal model stage. Several fatal problems still need to be solved for future clinical translation. Firstly, the issue of donor source needs strict regulation, which has failed to form a complete standardization and unification worldwide. Exosomes are under different regulatory models in different countries, in the United States exosomes are classified as drugs; in Europe and Japan, exosomes are classified as biologics (Wang H. et al., 2025). Secondly, as a new type of treatment, exosomes still need to be verified through more clinical trials in the future to see if they are effective in the long term and if there are potential side effects.

### 4.4 Hydrogels

Advantages of hydrogels for developing tissue scaffolds in DFU healing lie in their structural and functional properties. First, they possess a hydrophilic polymer chain network with three-dimensional cross-linking, closely resembling the natural ECM. This structural mimicry enables effective drug loading and sustained release. Additionally, the hydrogel’s ECM-like environment provides an optimal microenvironment for cellular activities essential to wound repair (Choudhury et al., 2017; Güiza-Argüello et al., 2022). Importantly, the hydrogel is also transparent, allowing real-time monitoring of the complex pathology of DFUs at different stages (Wang X. et al., 2022). Additionally, hydrogels can stop bleeding by adhesion and create a long-term suitably moist local environment for the wound. Also conform to host wound tissue, which prevents excessive inflammatory response and thus protects the wound from infection (Shi et al., 2020; Liang et al., 2021; Gorain et al., 2022).

**FIGURE 3:**
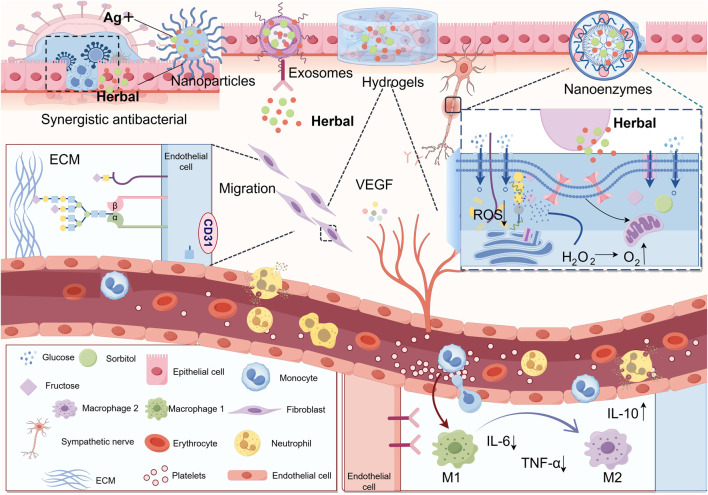
The integration of herbal active components with advanced nanomaterials significantly enhances therapeutic efficacy: (1) Synergistic combination with nanoparticles exhibits potent antibacterial activity by targeting and disrupting bacterial biofilms (2) Conjugation with nanozymes mimicking multienzyme functions enables glucose decomposition to alleviate hyperglycemic conditions, suppresses ROS accumulation to mitigate oxidative stress, and converts hydrogen peroxide into oxygen to improve ischemic-hypoxic wound microenvironments (3) Complexation with exosomes facilitates targeted delivery of herbal constituents, precisely promoting angiogenesis, fibroblast migration, and macrophage polarization regulation at injury sites (4) Incorporation into hydrogels leverages their transparency for real-time monitoring and adjustable release, while providing mechanical support and infection protection for DFU wounds (Thanks for Fig draw).

The combination of herbal active components with hydrogel can extend the action time of them and realize the advantages of “multi-target regulation-intelligent delivery-regulation of micro-environment-synergistic antimicrobial” with the help of advanced material properties of hydrogel. Zhang et al. prepared a composite hydrogel by loading Panax ginseng total saponin (PNS) on hyaluronic acid (HA)/carboxymethyl chitosan (CMCS). By establishing a skin wound model in type II diabetic SD rats, this hydrogel was found to significantly increase the wound healing rate in SD rats. The herbal hydrogel can not only reduce the inflammatory response of the body and reduce the expression of pro-inflammatory factors TNF-α and IL-6 but also enhance the expression of anti-inflammatory factor IL-10. It can also increase the expression of VEGF and play a role in promoting angiogenesis, which synergistically promotes the rapid healing of wounds (Zhang L. et al., 2023). Curcumin, possessing immunomodulatory and angiogenic properties, was formulated into a composite hydrogel. *In vivo* experiments demonstrated that this system not only scavenges ROS accumulation but also downregulates IL-1β expression while upregulating CD31 expression, thereby promoting angiogenesis and collagen deposition (Fan et al., 2024). In a recent study, a composite hydrogel with antimicrobial and antioxidant properties was made by ligating salvianolic acid B (SAB), which has anti-inflammatory properties and promotes local microcirculation, with Metal-polyphenol nanocomposite hydrogel. Its combined action synergistically promotes the healing of DFUs in an all-encompassing manner. On one hand, SAB and GOx act as anti-infective, microenvironmental modulators and pro-angiogenic agents, and on the other hand, they can also be released to improve mitochondrial energy metabolism through nanoparticles released in response to acidic wounding environments (Gong et al., 2025). Figure 3 summarizes the applications of active components of TCM in combination with novel delivery systems.

## 5 Conclusion and perspectives

This review adopts a molecular biology perspective to compare the mechanisms of normal wound healing *versus* impaired healing in DFUs. It elucidates the multi-target synergistic effects of herbal medicines and their formulations in DFU management, highlighting a therapeutic paradigm shift from passive debridement to active regeneration. Finally, the paper synthesizes emerging research trends, emphasizing the integration of herbal active components with novel delivery systems. This fusion underscores the promising applications of herbal medicine in interdisciplinary fields such as regenerative medicine and dermal tissue engineering.

Although herbal medicines have demonstrated the therapeutic advantages of multi-target regulation in the treatment of DFUs, which has shifted the healing of DFUs from “passive debridement” to “active regeneration”, there are still many challenges and opportunities in promoting the implementation of Chinese herbal medicine treatment at present. (1) Basic experiments: Existing experimental animal models are insufficient for further clinical translation. The current animal models are only centered around small rodents, which can simulate the pathological process of DFUs but in fact have differences in their human skin structure. In the future, it is necessary to overtake the research towards large animal models such as Ossabaw and Yorkshire pigs, which are highly homologous to human skin, as well as zebrafish juvenile models. In addition, mouse models of MDROs infection should be added, 3D-printed biological models should be developed to simulate DFUs biofilms, and the advantages of herbal medicines over antibiotics should be further evaluated. Future research should continue to explore novel, highly specific biomarkers for DFUs. Leveraging cutting-edge technologies such as single-cell RNA sequencing, multiparameter flow cytometry, and imaging mass cytometry, in-depth analysis of the dynamic DFU microenvironment must be conducted to identify new therapeutic targets and preventive strategies, thereby accelerating the advancement of precision medicine for DFUs. Additionally, ongoing efforts are needed to elucidate whether herbal medicines and their formulations can promote ulcer healing by influencing DFU biomarkers. These collective efforts will facilitate the translation of discoveries from bench to bedside. (2) Clinical trials: Existing clinical trials are mostly small-scale exploratory experiments, and it is recommended that large-scale, multicenter, prospective randomized controlled trials (RCTs) be rigorously designed. To make up for the lack of overall clinical trial data, and to solve the long-term dilemma of the failure of polyherbal therapies to form a widely recognized treatment guideline. In addition, there is a serious gap in long-term efficacy and safety data: currently, most patients are observed after 2–4 weeks of treatment, and it is recommended that systematic and long-term clinical follow-up be included in order to assess the rate of ulcer recurrence and the quality of post-treatment survival, as well as potential toxicity and other risk issues. In the future, a comprehensive assessment of the combined efficacy, safety, and patient prognosis of herbal medicines will provide reliable support for the clinical translation of herbal medicines for DFUs. (3) Quality and control of herbal medicines: Given the inherent complexity of herbal components, variability in extraction processes, and heterogeneity of composite drug delivery systems, we propose a comprehensive quality control framework: For raw herbal materials, standardized cultivation practices should be implemented from the source to eliminate heavy metal and pesticide contamination. Regarding multi-herbal formulations, chromatography and mass spectrometry techniques integrated with Quality Markers (Q-markers) must be adopted to establish quantitative compositional standards. Furthermore, efficacy biomarkers—such as fibroblast migration rate and macrophage polarization ratio—should be incorporated into quality specifications to enhance therapeutic outcomes for DFUs. For mechanistic validation, network pharmacology should be employed to construct “compound-target-disease pathway” models predicting therapeutic mechanisms, followed by rigorous *in vitro* and *in vivo* validation to scientifically elucidate the multi-target synergistic mechanisms of herbal formulations, ultimately establishing a standardized quality control system for herbal medicines formulations. (4) The future of novel delivery systems for herbal active components: although the combination of herbal medicines and novel delivery systems can achieve targeted delivery and sustained release deep into the ulcer, given that DFUs wounds are dynamically changing, the future should be designed to be dynamically responsive to the microenvironment of DFUs using PH-responsive, enzyme-responsive, and photo-thermal-responsive composite delivery systems. As well as glucose-sensitive delivery systems targeted at ameliorating peripheral vasculopathy, neuropathy, and smart insulin delivery in patients with DFUs. In recent years, it has been found that modulation of gut flora can affect DFU healing and responsive systems for gut targeting could be designed in the future. In addition, the combined application of 3D printing technology, carbon quantum dot technology, and herbal active ingredients in promoting wound healing for DFUs remains to be widely adopted. (5) Prevention and personalized treatment: Clinically, a stratified prevention system should be established. Type 1 and Type 2 diabetes have heterogeneous pathological foundations, with the latter being the primary conversion type for DFUs due to uncontrolled hyperglycemia. Given the preventable nature of Type 2 diabetes, emphasis should be placed on lifestyle interventions and glycemic control to enable early screening and disease progression interruption. Type 1 diabetes requires lifelong monitoring and complication prevention. The core of ulcer prevention lies in “zero-level prevention”—enhancing patients’ foot self-examination awareness through education and conducting regular systemic complication screenings to facilitate effective early intervention. Furthermore, treatment plans must be individualized: systemic factors (such as the presence of other chronic diseases, vascular disease, neuropathy, and infection), wound characteristics, and lifestyle habits vary among DFUs patients. Adopting a uniform treatment approach is unwise; instead, the key feature of personalized treatment is delivering the right therapy to the patient at the right time, rather than using all possible curative treatments. Integrating artificial intelligence (AI) and wearable devices enables dynamic monitoring and precise stage-based intervention, complemented by the immune-regulating and microcirculation-improving functions of TCM to reconstruct the body’s wound-healing capacity. On this basis, integrating global healthcare systems ensures that patients have no concerns regarding post-treatment care. (6) Comprehensive treatment strategy: In order to cope with DFUs, a chronic disease with extremely complex pathomechanisms and a lengthy course. It is urgent to promote a comprehensive treatment strategy. In a clinical trial of 100 patients with DFUs, treatment with ultrasonic debridement in combination with cortex phellodendri compound fluid showed a significant reduction in the size of the ulcers compared to the control group after 4 weeks of treatment and an overall treatment efficacy of up to 98 percent (Gao et al., 2022). A growing number of basic trials and clinical studies have revealed the therapeutic efficacy and economic advantages of herbal medicines; herbal therapies will be transformed from “empirical medicine” to “multi-targeted therapies with clear mechanisms.” Therefore, in the future, the overall advantages of herbal medicines treatment will be combined with modern medical methods to promote an integrated treatment strategy in order to provide better therapeutic effects and prognostic management for patients with DFUs.

In the future, we should advance the integration of herbal medicines with personalized treatment and precision medicine to accelerate breakthroughs in overcoming the bottlenecks in treating DFU wounds.
